# Magnetic Resonance Imaging or Ultrasound in Localized Intermediate- or High-Risk Soft Tissue Tumors of the Extremities (MUSTT): Final Results of a Prospective Comparative Trial

**DOI:** 10.3390/diagnostics12020411

**Published:** 2022-02-05

**Authors:** Bianca Bignotti, Federica Rossi, Alessio Signori, Nicola Solari, Bruno Spina, Carlo Martinoli, Alberto Stefano Tagliafico

**Affiliations:** 1 Ospedale Policlinico San Martino, 16132 Genoa, Italy; bianca.bignotti@hsanmartino.it (B.B.); nicola.solari@hsanmartino.it (N.S.); bruno.spina@hsanmartino.it (B.S.); 2 Department of Experimental Medicine (DIMES), University of Genoa, 16132 Genoa, Italy; federica.rossi@asl2.liguria.it; 3 Ospedale Santa Corona, Pietra Ligure, 17027 Genoa, Italy; 4 Department of Health Sciences (DISSAL), University of Genoa, 16132 Genoa, Italy; alessio.signori@unige.it

**Keywords:** sarcoma, ultrasound, magnetic resonance imaging, accuracy, recurrence

## Abstract

Objectives: To report final results of the MUSTT trial, which has been designed to independently compare magnetic resonance imaging (MRI) and ultrasound (US) for local recurrences of non-metastatic patients operated for malignant soft tissue tumors (STT). Methods: Magnetic resonance imaging or ultrasound in soft tissue tumors (MUSTT) is a prospective monocentric study recruiting asymptomatic, non-metastatic patients operated on for localized soft tissue sarcomas between 2015 and April. Eligible patients had MRI and physician-performed ultrasound (US) with an independent interpretation of imaging. Outcome measures were compared using ROC analysis and the X^2^ test. An analysis of all patients was performed on a per-follow-up event basis. Results: A total of n = 51 patients who met the inclusion criteria agreed to participate. Among them, n = 8 were lost to follow-up, n = 6 had US and MRI acquired after a time frame > 7 days and were therefore excluded. Complete data available for 37 patients with 232 MRI and 232 US scan were finally considered (men/women: 18/20; age range, 18–84 years). Recurrences within 5 years occurred in 10/37 patients (27%). ROC analysis comparing US and MRI showed an AUC with 95% confidence intervals of 0.909 (0.832 to 0.981) for US and 0.966 (0.939 to 0.989) for MRI with Prob > X^2^ = 0. Conclusions: Each of these tests detected local recurrences with suitable accuracy. MRI did not result clearly superior to US in terms of diagnostic accuracy, but US showed some false positive or negative results.

## 1. Introduction

Soft tissue tumors (STT) are represented by a wide range of different histological and molecular subsets with very low incidence populations at all ages [1]. STT represents the majority of sarcomas (soft tissue tumors ~75%, gastrointestinal ~15%, and ~10% bone sarcomas) [2]. The prognosis of STT is influenced by different factors such as grading, resection margin, location, age >64 years, and distant sarcoma metastasis [1]. However, local recurrences (LRs) not only influence tumor control locally but also result to influence overall survival in several investigations [3,4,5]. The rate of LRs has been reported to vary from 8.5% after 24 months to 20–32% after 10 years [6]. The risk of LRs is higher in the first years after surgery, and it has been estimated that 60% to 70% of recurrences occur within 2 years and >90% occur by 5 years [7]. Radiotherapy reduces LRs rates [8]. Although some studies showed that the survival rate of patients with STT, especially those with high-risk lesions, could be improved by consistent local follow-ups with imaging, there are not currently accepted evidence-based consensus guidelines on how and to what extent regular follow-up imaging influences the outcome of these patients [9]. In standard radiological clinical practice, both ultrasound (US) and magnetic resonance imaging (MRI) are currently used to rule out a recurrence in patients operated on for STT [1,2,10,11,12]. It is widely accepted that US is highly accessible, radiation-free, easily repeatable, fast, and with a spatial resolution higher than MRI when very-high-frequency transducers evaluate superficial tissues [10]. According to guidelines, STTs should be managed in tertiary sarcoma centers; however, radiologists can be involved in follow-up strategies even outside tertiary centers for practical reasons [2,10,12]. Following 2018 ESMO (European Society of Medical Oncology) guidelines on follow-up imaging of STT, there is no clear evidence to indicate the optimal routine follow-up of surgically treated patients with localized disease nor on the use of US and/or MRI or CT to detect LRs [2]. Recent retrospective studies found encouraging results regarding the performance of US in the detection of LRs in patients with localized soft tissue sarcomas of the limb, which resulted in having 88% sensitivity and 94% specificity [13]. However, there is a need for prospective studies because a practical approach is still in place at several institutions with frequent follow-up even every 3–4 months in the first 2–3 years for intermediate-high-risk lesions [2,10,12]. Therefore, considering that performing US is easier and undoubtedly less stressful for patients than having a high-field MR (1.5T or 3.0T as suggested by guidelines), the purpose of the study was to prospectively compare MRI and US in the detection of local recurrences of adults patients with localized intermediate/high-risk soft tissue tumors of the limbs.

## 2. Materials and Methods

Magnetic resonance imaging or ultrasound in soft tissue tumors (MUSTT) was implemented as a prospective monocentric study recruiting asymptomatic, non-metastatic patients operated on for localized soft tissue sarcomas. Our aim was to compare the detection of LRs using widely adopted imaging methods such as US and MRI that allow independent test interpretation. The study received IRB approval (304REG2015), and the patients involved provided written informed consent. MUSTT is a registered study (ClinicalTrials.gov Identifier: NCT02834585) sponsored by the University of Blind City for Review and by the Blinded for Review.

### 2.1. Patients

Patients with STT who met the inclusion criteria were prospectively included and studied with US and MRI in our center, which is a regional referral center for diagnosis and treatment of STT and followed-up every 3–4 months. Patients to be included had to meet the following inclusion criteria:

- Age > 18 years;
- Surgery for proven primary intermediate or high-risk STT of the upper or lower limb (e.g., spindle-cell or pleomorphic histology, myxoid-round cell liposarcoma, leiomyosarcoma, synovial sarcoma, malignant peripheral nerve sheath tumor, undifferentiated pleomorphic sarcoma, myxofibrosarcoma, pleomorphic liposarcoma, pleomorphic rhabdomyosarcoma, et al.) according to WHO criteria;
- Localized disease (non-metastatic);
- ECOG performance status < 1;
- Signed informed consent;
- Complete US and MRI follow-up every 3–4 months;
- Follow-up of 24 months for negative US and MRI findings;
- Time-lapse between US and MRI < 7 days.

Exclusion criteria were the following:

- Pregnancy or lactation;
- Other malignancies within the past 5 years;
- Metastatic disease;
- Low-risk sarcomas;
- Lack of informed consent or limited compliance;
- Impossibility of warranting imaging follow-up at the center;
- Absence of definitive histological diagnosis on surgically excised masses (when surgery was not performed or performed in other centers).

### 2.2. Imaging Follow-Up Program

The post-operative to detect LRs imaging was planned with US and MR imaging every 4 months for the first 5 years and follow-up of 24 months for negative US and MRI findings after 5 years. Clinical assessment, chest CT and relevant assessment were performed to exclude metastatic disease as suggested by current protocols [2].

### 2.3. Ultrasound

All US examinations of upper and lower limbs were performed by a team of 4 experienced radiologists (minimum experience: 6 years; maximum experience 27 years) with specific expertise in musculoskeletal imaging and soft-tissue masses as suggested by the Tumor Subcommittee of the European Society of Musculoskeletal Radiology (ESSR) [10,14]. Ultrasound probes for limb evaluation to rule out the presence of LRs included last-generation broadband linear array transducers (at least 13 MHz of frequency) of different vendors (Esaote My Lab, different versions, and Canon Aplio 800). Standard US evaluation included in the radiological report: anatomical location of the lesion with a clear description of anatomical relationships among the lesion, the muscles, the nerves, and the vessels, intra- or intermuscular location and compartmental involvement; size, pattern of growth, relation to the fascia (superficial or deep), color-Doppler evaluation, presence or absence of intra-lesion necrosis, bleeding, posterior acoustic enhancement/shadowing, suspected calcifications, shape, borders/margins. Finally, the radiologist had to conclude if the US examination was consistent with LR or not on the basis of ESSR criteria and personal judgment [10]. In the case of possible LR at US, further biopsy was warranted.

### 2.4. Magnetic Resonance Imaging

Standard musculoskeletal limb MRI was performed on different vendor 1.5T (Siemens Magentom Aera 1.5T or Magnetom Avanto1.5T) or 3T scanners (Siemens MAGNETOM Prisma 3.0T) with anatomical T1-weighted sequences, T2-weighted sequences with and without fat saturation, and T1-weighted sequences with fat saturation before and after intravenous administration of gadolinium chelates as per ACR guidelines (https://www.acr.org/ accessed on 17 June 2021). MRI planes and coils were tailored on the anatomical region with at least 4 mm of slice thickness [15,16,17]. Coils used were: 4-channel flex coils of different sizes for the extremities combined, when necessary, with 8- to 32-channel phased-array body coils included and Tx/Rx Knee 15 Flare Coil for the knees. The MRI protocol started from the following parameters slightly adapted to the region considered: slice thickness at least 4 mm, slice spacing = 1 mm, matrix size 384xT1-weighted MR imaging repetition time/echo time (TR/TE) 500 ms/8 ms; acquisition voxel size (mm^3^) 0.6 × 0.7 × 3.0; T2-weighted MR imaging TR/TE 6200 ms/110 ms; acquisition voxel size (mm^3^) 0.6 × 0.7 × 3.0; T1-weighted MR imaging with gadolinium TR/TE = 500/12 ms; acquisition voxel size (mm^3^) 0.6 × 0.7 × 3.0; T2-weighted MR imaging and T1-weighted MR imaging with gadolinium is acquired with fat saturation. As for US, the radiologist had to state if MR was consistent with LR or not according to current guidelines [10]. Each patient included at every follow-up had both US and MRI acquired and reported within 7 days by different radiologists independently. Radiologists were blinded to the US findings if they were reporting the MRI and vice versa. The radiologist who performed US did not perform MRI and vice versa to guarantee independent reading of US and MRI. Radiological findings included in the MRI report to detect recurrences: location, three-dimensional size, morphology, shape, borders, relation to the superficial fascia, intra- extra- compartmental location, relation to adjacent structures (vessels, nerves, joints, …), and surrounding tissue alterations, distance to an external landmark, satellites, multiplicity, loco-regional lymph nodes. The signal intensity of the lesions (e.g., presence of fat, hypo-intensity on fluid-sensitive sequences), homogeneity (esp. heterogeneity of >50% of the tumor volume on fluid-sensitive fat-suppressed images), diffusion restriction (if available, with ADC), vascularity, and enhancement. The presence of a possible recurrence was defined according to ESSR guidelines [10] and then confirmed or excluded by biopsy.

To assess the diagnostic performance of US and MR on a per-event and per-lesion basis, histology and surgery were used as a reference standard for US, and MRI reported as positive (true positive). The false positive for US or MRI was defined when histology was negative after a positive imaging evaluation. The US or MRI false negative was assigned if LRs appeared before the next scheduled follow-up following literature suggestions [4,18]. No indeterminate US and MRI reports were allowed for the purpose of this study.

### 2.5. Statistical Analysis

US and MRI reports were compared against the reference standard (histology, surgery, or follow-up) on a per-even and per-lesion basis for each follow-up event. ROC curves for each modality and in combination were calculated. In addition, sensitivity and specificity with 95% confidence intervals (CIs) were estimated. ROC curves and standard diagnostic performance indicators were also estimated (positive predictive value (PPV), negative predictive value (NPV), the likelihood ratio for positive results (LH+), accuracy, and post-test probability (post-P). A *p*-value < 0.05 (2-sided) was considered statistically significant. Logistic multiple regression was performed to exclude or identify the effect of independent variables (e.g., patient age, sex, reporting radiologist) on diagnostic accuracy. Commercially available software (MedCalc Software Ltd. Diagnostic test evaluation calculator. https://www.medcalc.org/calc/diagnostic_test.php (Version 20.009; accessed 2 July 2021, SPSS version 14, Chicago, Ill and STATA 17) were used for analysis.

### 2.6. Power Analysis

Power analysis and sample size calculation were performed considering a 1% difference in the incidence of the lesion between US and MRI. Indeed, an incidence of the event (recurrences) of 21/232 (9%) and 24/232 (10%) for US and MRI was present. The two study groups were considered to receive independent imaging evaluation with a binomial primary endpoint (LR or not). Considering an enrolment ratio of 1 (equal enrolment for US and MRI), the desired alpha (probability of type I error) of 0.05, beta (type II error) of 0.2, and a power (1-beta) of 0.8, a sample size of n = 12,208 was calculated to have a significant difference among two groups. This number has been considered clinically not relevant; therefore, this study was considered sufficiently powered [15,16,17,19,20].

## 3. Results

A total of n = 51 patients who met the inclusion criteria agreed to participate. Among them, n = 8 were lost to follow-up, n = 6 had US and MRI acquired after a time frame > 7 days and were therefore excluded. Complete data available for 37 patients with 232 MRI and 232 US scans were finally considered for the analysis. From 37 patients with the complete US and MR imaging follow-up examination (men/women: 18/20; age range, 18–84 years), histology and clinical characteristics are reported in Table 1. After surgery for the primary tumor resection, margins were declared clear (R0) by the surgical team in 20/37 (54%), whereas in 17/37 patients (46%), microscopically involved margins (R1) were registered. Recurrences within 5 years occurred in 10/37 patients (27%): n = 4 liposarcomas, n = 4 sarcomas, 2 synovial, 2 myxoid, n = 1 leiomyosarcoma, n = 1 atypical adipose tumor.

**Figure 1:**
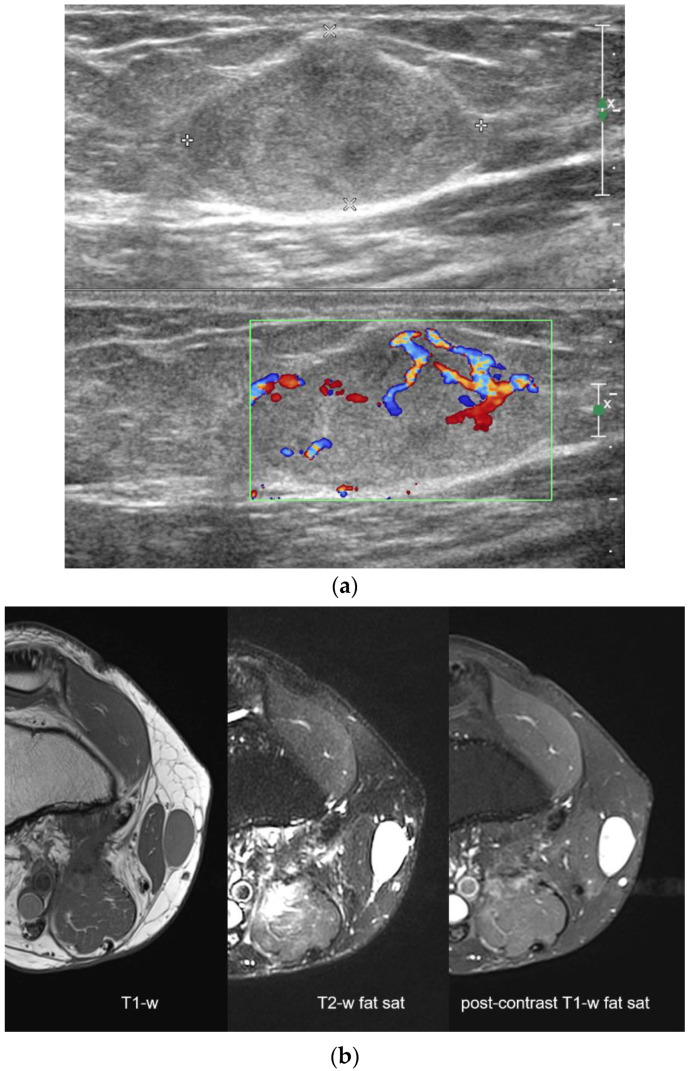
A 38-year-old man operated for a localized liposarcoma of the popliteal fossa. Images obtained after one year show a recurrence. Ultrasound images obtained at the medial aspect of the knee show a hyperechoic nodule (**a**) with intralesional color-Doppler signals and some slightly hypoechoic intralesional areas. MRI images (**b**) show a nodule isointense on T1-weighted sequences and hyperintense on both T2-weighted sequences with fat saturation and post-contrast T1-weighted sequences. Both US and MRI were reported as recurrence.

**Figure 2:**
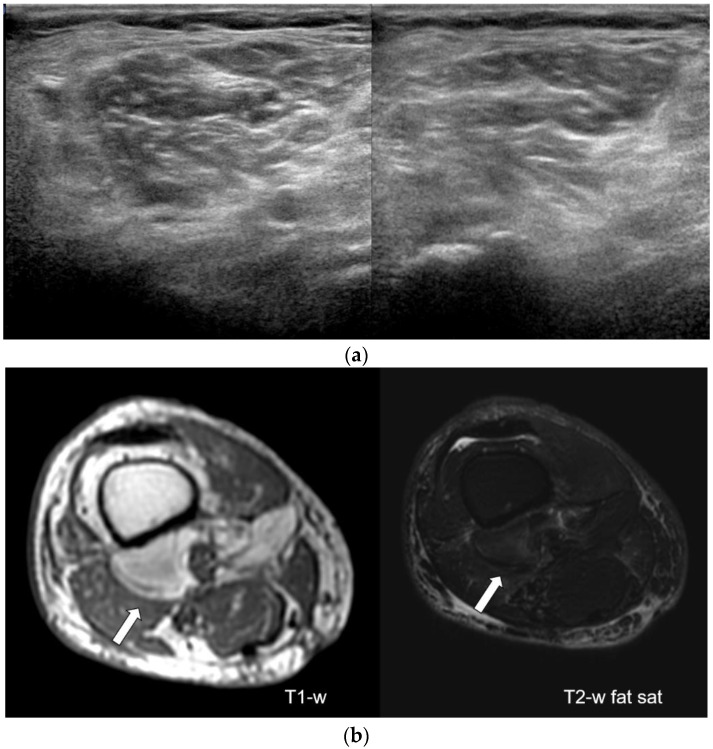
A 78-year-old man operated for a localized liposarcoma of the popliteal fossa in follow-up ultrasound images (**a**) obtained at the popliteal fossa were reported as negative for local recurrence with no recurrence demonstrated in the follow-up. MRI images (**b**) show a deep-located (white arrow) nodular lesion with MRI signal consistent with fat on T1-weighted sequences and T2-weighted sequences with fat saturation. A slight hyperintensity on T2-weighted sequences with fat saturation was present. In this case, ultrasound was reported negative for recurrence, whereas MRI was reported positive.

**Figure 3:**
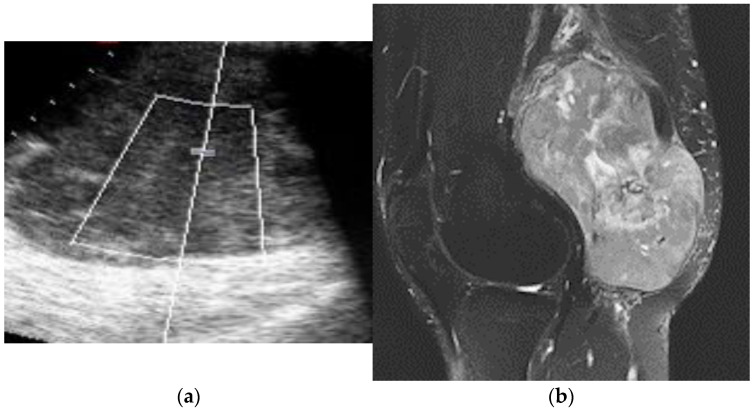

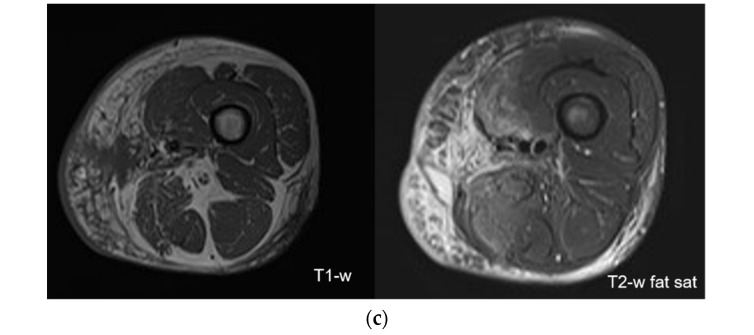
A 45-year-old man operated for a large MPNST with translocation t(X; 18) (p 11.2; q 11.2) and of genes SYT/SSX of the popliteal fossa shown by both US (**a**) and MRI (**b**). Follow-up did not show any changes consistent with relapse; only minor edema and fluid collection were present (**c**). Both MRI and US were reported negative for recurrence.

**Figure 4:**
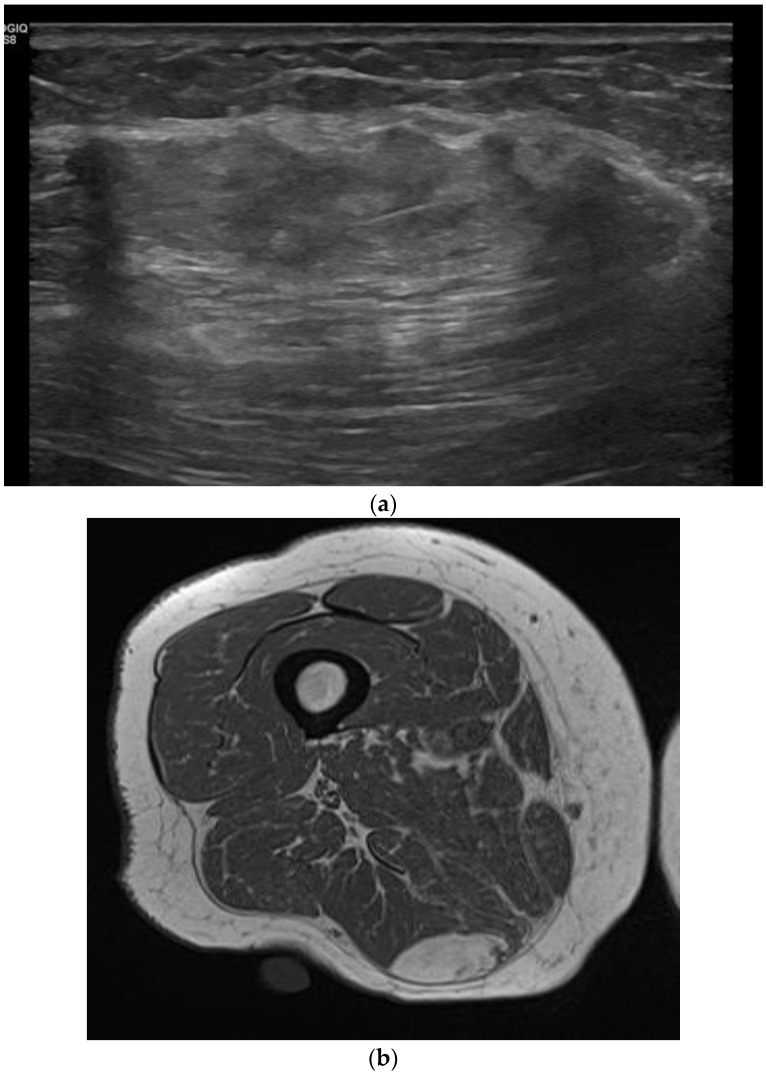

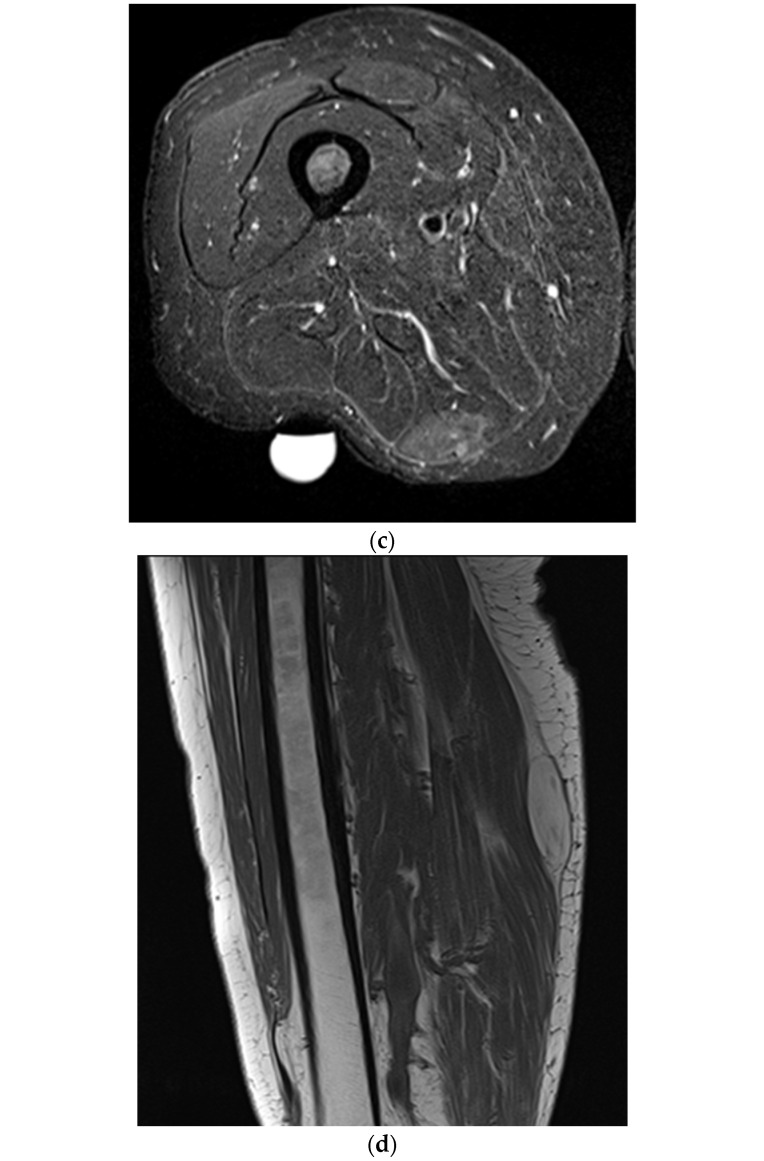
A 79-year-old woman operated on for a high-grade liposarcoma. At follow-up, both US (**a**) and MRI (**b**–**d**) detected a solid nodule in the posterior aspect of the thigh with prevalent fatty content near the surgical area. Both US and MRI were reported as consistent with recurrence; however, pathology did not show any malignant tissue after surgery.

**Figure 5:**
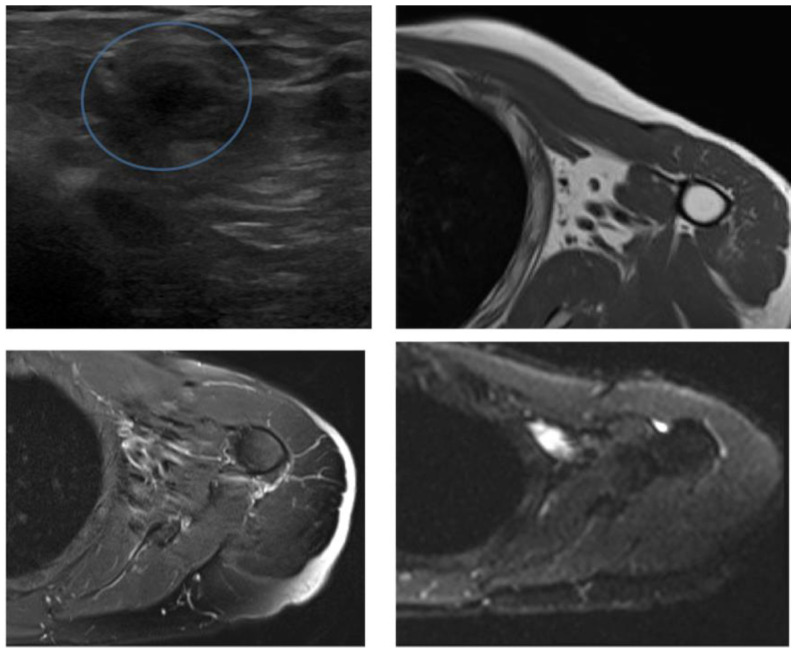
A 71-year-old man with intermediate-grade leiomyosarcoma of the proximal arm. US was reported as positive for recurrence due to the detection of a pseudomass/irregularity of the superficial tissues of the proximal arm (circle). MRI did not show any mass. Follow-up did not show any recurrence.

**Figure 6:**
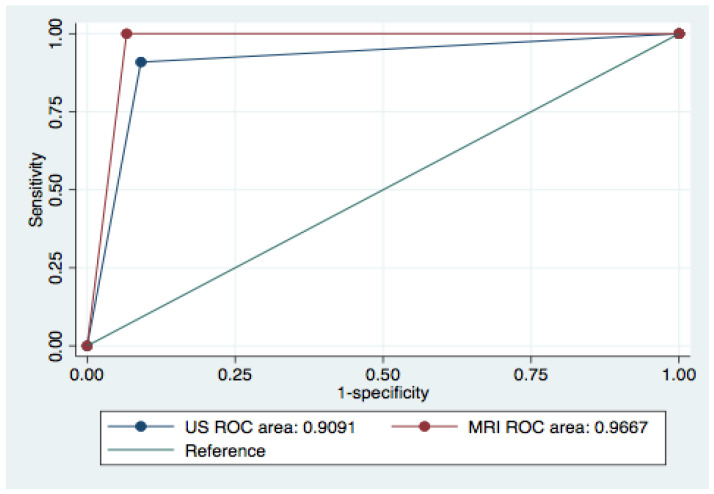
ROC analysis of US and MR imaging follow-up.

The median number of follow-up events per patient was 8 (range 6–24). Examples of US and MRI cases are illustrated in Figure 1, Figure 2, Figure 3, Figure 4 and Figure 5. ROC analysis comparing US and MRI showed an AUC with 95% confidence intervals of 0.909 (0.832 to 0.981) for US and 0.966 (0.939 to 0.989) for MRI with X^2^ = 0.0766 (Figure 6). Details for diagnostic performance of US and MRI are reported in Table 2.

## 4. Discussion

According to recent guidelines on STT imaging and follow-up, we tried to fill the gap regarding the need to have prospective studies formally comparing US and MRI in the follow-up of patients operated for localized intermediate or high-risk soft tissue tumors of the extremities [2,10,18]. We designed a comparative study where the two imaging techniques used more often to rule out LR, US, and MRI, were compared in the same patient. The study design included independent radiological evaluation of US and MRI to overcome the need for a randomized trial that is unfeasible for ethical reasons, and to reduce the number of patients to be included as already performed in other imaging studies [21,22,23,24,25]. To increase the reliability of this approach and reduce biases, we allowed a < 7 days of time frame between US and MRI, limiting as much as possible to have both US and MRI in the same session. On the other hand, we did not choose a randomized comparative trial to avoid offering patients a potentially worst radiological technique than the alternative. In other words, this study design allowed patients to receive the best imaging follow-up available so far. Results of this trial show that both US and MRI are highly sensitive in detecting LRs in this group of patients with localized disease, although overall accuracy of MRI resulted slightly higher: AUC for US was 0.909 (95% C.I. = 0.832 to 0.981) for US and AUC for MRI 0.966 (95% C.I. = 0.939 to 0.989) for MRI. When comparing US and MRI performances, there was no formal statistical difference between the two techniques if X^2^ statistics was used considering the 232 events that were included in the analysis. The present study included 37 patients: adding more patients and follow-up events could have increased the chances of having a statistically significant result in favor of MR, but it is not sure that the result would have been clinically meaningful. The results of this prospective study seem to confirm the results obtained in a retrospective analysis focused on the performance of ultrasound [13]. Indeed, US resulted in being highly specific for LRs detection with few false positives due to the presence of scarring and granulation tissue that resembled a mass lesion. Using ultrasound probes with low frequencies, 5 MHz to 7.5 MHz, which are different from up-to-date broadband linear array probes and selecting a few patients Choi et al., in 1991, stated that the accuracy of US for the detection of local recurrence was similar to that of MRI. On 26 patients, they reported 100% sensitivity and 79% specificity for US and 83% and 93%, respectively, for MRI [26,27]. In 1993, an Italian research group from the National Institute for Cancer Research of Genova led by Pino et al. [27], evaluating the same number of patients of Choi et al. [26], reported a sensitivity of 87% for US, compared with 69.6% for computed tomography for lesions up to 5 mm. Arya et al. [28], in 2000, evaluated with US 50 patients operated for sarcoma surgery finding 26 recurrences, 18 non-recurrences, 4 benign diseases, and 2 indeterminate lesions reporting a sensitivity for US of 92.3% and a specificity of 94.4%.

US has obvious advantages of cost efficiency compared to MRI, but US, especially in the post-surgical setting, has the advantage of not being subject to artifacts from metallic hardware and to potentially guide drainage (and obviously biopsy) [29]. Our surveillance protocol can be considered intense, and regarding the use of MRI or US, we cannot affirm that one technique is clearly better than the other; however, the overall performance of MRI resulted in being slightly better than that of US. In the present study, US resulted in being less effective in detecting deep lesions, for example, at the popliteal fossa for lesions containing fat. This is one of the few studies with prospective data helping to address an unmet need in clinical follow-up of patients with STT of the extremities [2,10,12,18]. Differently from the work by Singer et al. [30], in our study, the reference standard was histology, surgery, or follow-up for both MRI and US. Singer et al. [30] considered a subsequent MRI as a possible reference standard for US, therefore, introducing a bias related to the reference standard.

Compared to the study of Singer et al. [30] and Tagliafico et al. [11,13], our results in terms of accuracy could be considered overlapping in spite of slightly different study designs. In this study, AUC for US was 0.909 (91%), whereas, in the study of Singer et al. [30], the accuracy was 92.6%, and the AUC for MRI was 0.966 (96%) compared to 97.6% in the study of Singer et al. [30].

This study, although it is one of the few prospective studies directly comparing US and MRI in the same patient with the surgical and histological reference standards, has some limitations. Due to the relative rarity of STT and to the necessity of having strict protocol adherence (see inclusion criteria), the study cohort could be considered moderate in number. However, this study cohort is comparable to previous studies, and it was evaluated with a better study design [13,30].

US is known to have reduced performance in deeply located lesions, and it is normally considered operator dependent. Indeed, the STTs analyzed in this study are mostly of the upper limb. In the case of a deeply located lower limb tumor, it is possible that the referring physician did not send the patients for US or MRI in this center due to the known limitations of US in deeply located lesions. In this study, radiologists were highly experienced in US usage, and we do not know if these US performances are clearly reproducible at other institutions and to what extent. However, there are many efforts in the scientific community to develop guidelines and to standardize US usage among different specialists to have reliable and repeatable US examinations [10,12,31,32,33,34]. Against the usage of MR, a study by there is a study by Labarre D et al. [35] stated that the systematic use of MR was not useful to detect asymptomatic local recurrences. However, MR is still considered the technique of choice for follow-up in spite of suitable and comparable performances of US. We would like to clarify that it is extremely probable that US performed in non-specialized centers by different operators would not be able to achieve these levels of reliability compared to MRI. Further larger prospective studies are needed to further clarify this issue.

In conclusion, this prospective study compared prospectively and with independent reading US and MRI in patients with localized intermediate or high-risk soft tissue tumors of the extremities. Each of these tests detected local recurrences with suitable accuracy. Incorporation of US in surveillance algorithms of patients with STT of the extremity STS have several advantages (even psychological) and could be discussed in guidelines, but the level of reliability of MRI is extremely high. It has still to be demonstrated that US performed in non-specialized centers by different operators is not inferior to MRI.

## Figures and Tables

**Table 1:** Summary of patient’s characteristics.

	All Patients (n = 37)
Age at diagnosis, years (median (range))	55 (18–86)
Sex	
Female	19
Male	18
Primary tumor size, mm (median (range))	55 (7–200)
Positive/negative resection margins (R1/R0)	17/20
Recurrence tumor size mm (median (range))	23 (7–53)
Number of patients with recurrences within 5 years	10
Number of patients with multiple recurrences	6
Primary tumor location	
Upper limb	27
Lower limb	10
Superficial location to superficial fascia of the recurrence	3
Deep location to superficial fascia of the recurrence	7
Histologic subtypes	
Synovial sarcoma	8
Nerve sheath tumors °	3
Myxofibrosarcoma	6
Leiomyosarcoma	4
Myxoid liposarcoma	6
Pleomorphic sarcoma	4
Other *	6

° MPNST: malignant peripheral nerve sheath tumor; Other *: 1(angiosarcoma epithelioid); 2(atypical adipose tumor); 1(extraskeletal osteogenic sarcoma); 1(angiomatoid fibrous histiocytoma); 1(hemangiopericytoma).

**Table 2:** (**a**) Summary of diagnostic accuracy parameters for US and MRI. (**b**) 2 × 2 cross-tabulation with number of positive and negative scans. TP = true positive, TN, true negative, FN = false negative, FP = false positive. (*) These values are dependent on disease prevalence.

(**a**)		
**US**		
**Statistic**	**Value**	**95% CI**
Sensitivity	91.30%	71.96% to 98.93%
Specificity	91.18%	85.86% to 94.98%
Positive Likelihood Ratio	10.35	6.28 to 17.05
Negative Likelihood Ratio	0.10	0.03 to 0.36
Negative Predictive Value (*)	98.73%	95.37% to 99.66%
Positive Predictive Value (*)	58.33%	45.94% to 69.76%
**MRI**		
**Statistic**	**Value**	**95% CI**
Sensitivity	100.00%	85.75% to 100.00%
Specificity	94.48%	89.78% to 97.44%
Positive Likelihood Ratio	18.11	9.60 to 34.18
Negative Likelihood Ratio	0.00	
Negative Predictive Value (*)	100.00%	
Positive Predictive Value (*)	72.73%	58.56% to 83.42%
(**b**) **2 × 2 cross-tabulation of US vs. MRI. X^2^ *p*-value: 0.0766.**		
	**US +**	**US −**
MR +	31	3
MR −	0	198
**NB: No true positive and false positive included.**		
**US**	**MRI**	
21	24	TP
194	199	TN
2	0	FN
15	9	FP

## Data Availability

The data presented in this study are available on request from the corresponding author.

## Abbreviations

STT	Soft tissue tumors
US	Ultrasound
MRI	Magnetic resonance imaging
ROC	Receiver operating characteristic
ESMO	European Society of Medical Oncology
ESSR	European Society of Musculoskeletal Radiology
WHO	World Health Organization
ECOG	Eastern Cooperative Oncology Group
ESSR	European Society of Musculoskeletal Radiology
ACR	American College of Radiology
AUC	Area under the curve
